# A Real-Time Four-Dimensional Reconstruction Algorithm of Cine-Magnetic Resonance Imaging (Cine-MRI) Using Deep Learning

**DOI:** 10.7759/cureus.22826

**Published:** 2022-03-03

**Authors:** Yuto Tamura, Kazuyuki Demachi, Hiroshi Igaki, Hiroyuki Okamoto, Masahiro Nakano

**Affiliations:** 1 Radiation therapy, Daiwa Institute of Research Ltd., Tokyo, JPN; 2 Nuclear Engineering, University of Tokyo, Tokyo, JPN; 3 Radiation Oncology, National Cancer Center Hospital, Tokyo, JPN; 4 Radiation Oncology, Cancer Institute Hospital Ariake, Tokyo, JPN

**Keywords:** long short-term memory, generative adversarial network (gan), optical flow, neural network, mridian, mr-linac, radiation therapy

## Abstract

Purpose

The purpose of this study is to propose algorithms and methods for achieving high accuracy in tracking and interception irradiation technology for tumors that move by respiration using MR-linac (MRIdian®, ViewRay Inc.) and to use deep learning to predict the movement of moving tumors in real time during radiation therapy and reconstruct cine magnetic resonance imaging (cine-MRI) into four-dimensional (4D) movies.

Methods

In this study, we propose a reconstruction algorithm using 4DCT for treatment planning taken before irradiation as training data in consideration of the actual treatment flow. In the algorithm, two neural networks made before treatment are used to reconstruct 4D movies that predict tumor movement in real time during treatment. Cycle GAN (generative adversarial network) was used to convert MR images to CT images, and long short-term memory was used to convert cine-MRI to 4D movies and predict tumor movement.

Results

We succeeded in predicting the time including the imaging time of the MR images, the lag until irradiation, and the calculation time in the algorithm. In addition, the conversion and prediction results at each phase of reconstruction were generally good so that they could be clinically applied.

Conclusions

The reconstruction algorithm proposed in this study enables high-precision radiotherapy while predicting the volume information of the tumor and the actual tumor position, which could not be obtained during radiotherapy.

## Introduction

Research background

In recent years, image-guided technology has been used as a standard position matching method in high-precision radiotherapy, and the results of radiotherapy have improved. Until now, image-guided radiotherapy (IGRT) has been mainly performed by cone-beam computed tomography (CBCT) or electronic portal image detector (EPID) installed in radiotherapy equipment [1,2]. While CBCT can directly and accurately visualize tumors in three dimensions with a tomographic image, EPID is characterized by being able to be observed in real time during irradiation by fluoroscopy. However, IGRT technology, which combines the advantages of both "direct 3D tumor visualization" and "real-time observation", was first put to practical use by MRI-linac (MRIdian®, ViewRay Inc., Oakwood Village, OH) equipped with an MR image guided device [3-5]. Real-time observation of tumors is also very effective for radiation therapy for lung cancer, which moves significantly with breathing. The purpose of this study is to realize high-precision radiotherapy for moving tumors by MRIdian.

The cine magnetic resonance imaging (cine-MRI) installed in the MRIdian draws the tumor during radiation therapy, and this technology may realize markerless radiation therapy [6,7]. However, it is only a grasp of the sagittal section, and as a problem of tracking and gating for irradiation of a moving tumor, there is a deviation of the irradiation position due to a time delay (system delay by irradiation device and control of multi-leaf collimator until irradiation). MRIdian has the imaging time of about 0.25 seconds per frame and an irradiation delay of about 0.5 seconds. In other words, when the tumor is confirmed on the monitor and irradiated, it is actually irradiated around 1.5 seconds ahead.

Objectives

Based on the research background shown above, this research’s objectives and functional requirements to achieve them are defined below. We propose algorithms and methods for achieving high accuracy of tracking and gating irradiation technology for mobile tumors using MR-linac. In order to achieve this objective with the current MR-linac device, three main functions are required. The first is the conversion of MRI to CT images while maintaining high contrast. The second is the estimating tumor three-dimensional (3D) movement by breathing and reconstructing 3D movies from cine-MRI. The third is the future prediction of tumor migration amount for MRI imaging time and device delay time.

## Materials and methods

Materials

The data analyzed in this study are the data provided by the National Cancer Center Hospital. Table 1 shows the patient data and modality information used. All are patients with tumors in the lungs who have undergone radiation therapy.

**Table 1:** Lung cancer patient data verified for reconstruction 4DCT, four-dimensional CT

Patient number	Age	Gender	4DCT	Cine-MRI and 3D MRI
1	85	Male	Aquilion LB (Canon Medical Systems)	MRIdian (ViewRay)
2	83	Male		
3	73	Male		
4	77	Female		
5	78	Female		

The CT/MR images of patients 1-4 were used as training data for cycle generative adversarial network (cycle GAN), and the data for patient 5 were used as training data for predicting the future of 3D movement from two-dimensional (2D) continuous image pixel values in long short-term memory (LSTM). Also, by interpolating the data of patient 5 in the time direction as the verification data for the entire algorithm (reconstruction from cine-MRI to 4D MRI), the processed data with the respiratory cycle and amplitude changed was used. All MRI images used were T1-weighted images.

Methods

Algorithm

**Figure 1:**
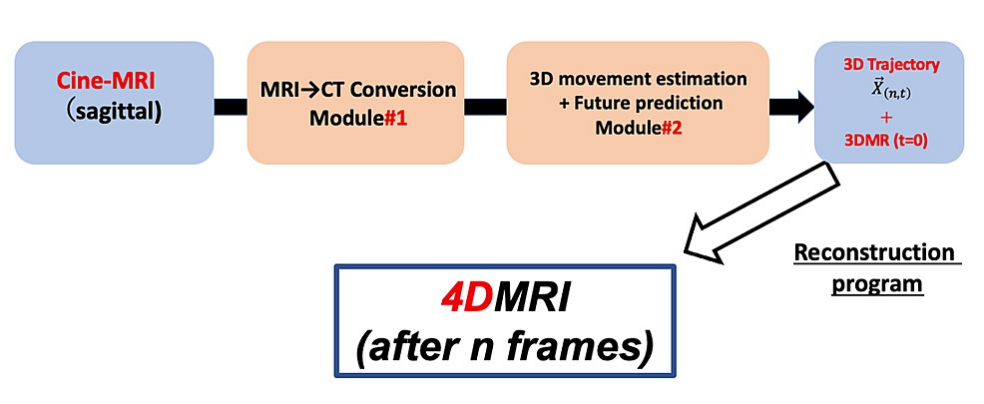
Real-time 4D MRI reconstruction algorithm during radiation therapy. Image credits: Yuto Tamura

In order to achieve the three functions necessary to achieve the purpose of this study, we decided to create two types of neural networks in advance before treatment and adapt them during treatment. Figure 1 shows the flow of reconstruction performed in real time during treatment.

The algorithm from the decision to conduct radiation therapy to the treatment date is shown. We obtain 4DCT of this 3D image around the irradiation area taken at 0.5-second intervals to create a radiotherapy plan. Using this 4DCT, the time series data of the 3D optical flow (OF) of N pixels is calculated, and this is used as the output data of the neural network. Also, considering the cine-MRI obtained during the treatment, the pixel value of each frame was acquired using the 2D cross-section movie cut out from the 4DCBCT movie taken before this treatment, and this was used as the input data of the neural network. The neural network that estimates the 3D movement amount from this CT image pixel value is Module#2 of Figure 1. Also, since the 3D movie for treatment planning is 4DCT, the input of Module#2 uses the pixel value of the CT image. However, since cine-MRI is obtained during treatment, we will create a neural network that converts MR images into CT images as Module#1.

**Figure 2:**
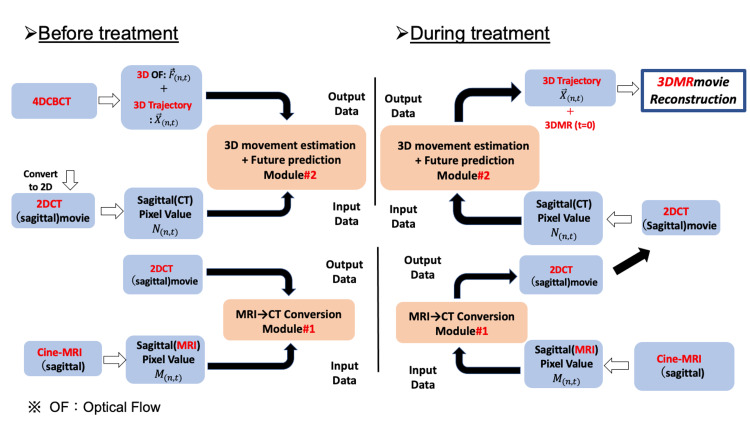
Overview of the algorithm before and during treatment. Image credits: Yuto Tamura

During treatment, the cine-MRI obtained in real time is converted into cine-CT by Module#1, and the 3D movement amount several frames ahead is estimated by Module#2. After that, 4D MRI is created by the 3D reconstruction program developed by us. Figure 2 shows the overview of the algorithm before and during treatment.

Module

Module#1 uses cycle GAN, which is an extended model of GAN [8]. Since normal GAN is supervised learning, a large number of accurately paired images of CT images and MR images are required. However, it is difficult to prepare a large number of training pair images of medical images with different modalities (such as MRI and CT images). Therefore, the proposed algorithm used cycle GAN, which enables unsupervised learning [9,10]. For learning, CT and MRI of patient 1-4 were used, and 760 tumor sagittal cross-sectional images were used. At this time, the CT image and the MR image do not have to have the same cross-section.

Module#2 solves the regression problem of estimating the 3D movement amount from the 2D MRI pixel values and, at the same time, constructs a network by LSTM to eliminate the deviation of the irradiation position due to the imaging time of cine-MRI and the device delay [11,12]. This made it possible to predict the respiratory cycle from the movement before and after the frame of interest. OF by the Lucas-Kanade method was used to calculate the amount of movement in 3D [13,14]. As a result, the amount of movement of each pixel around the tumor was vectorized in 3D. Then, 4D MRI is reconstructed by the reconstruction program from the 3D OF and the 3D MRI image taken by MRIdian immediately before the treatment.

## Results

In this section, in addition to the verification of each functional requirement defined for objectives of this research, we verified the reconstruction accuracy from cine-MRI to 4D MRI, high speed (real time), and adaptability to tracking and interception irradiation throughout the algorithm.

CT and MR images of patients 1-4 are used as training data for cycle GAN, and the data of patient 5 are used as training data for 3D movement future prediction from 2D continuous image pixel values in LSTM. In addition, by interpolating the data of patient 5 in the time direction as the verification data for the entire algorithm (reconstruction from cine-MRI to 4D MRI), the processed data with the respiratory cycle and amplitude changed was used. All MRI images used were T1-weighted images.

Results from conversion to CT image to reconstruction

In this study, considering the delay time from acquisition of cine-MRI to 4D MRI reconstruction and irradiation of the tumor area, 3D movement amount six frames (1.5 seconds) ahead is predicted from cine-MRI.

Considering that the movement of the tumor with breathing varies greatly for each patient, the learning model is created from the treatment plan 4DCT for each patient. We also created a learning set with a respiratory cycle (from maximum inspiration to maximum expiration) of 4.0 to 6.0 seconds to accommodate changes in the respiratory cycle during 4DCT imaging and during treatment.

**Figure 3:**
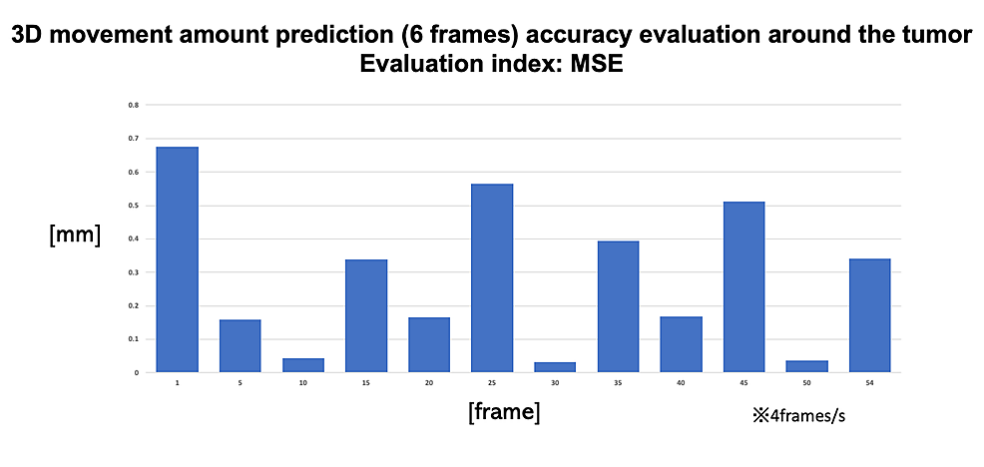
Three-dimensional movement amount prediction accuracy evaluation around the tumor.

Figure 3 shows the result of evaluating the error between the predicted value of the movement amount of each pixel in the tumor area and the correct answer data by OF on three axes.

From the results, it generally achieves to predict the amount of movement in three dimensions, and it will be possible to perform tracking irradiation within a set margin. In addition, the prediction error especially at the time of maximum inspiration is very small, and even higher accuracy irradiation can be performed by interception irradiation. There was also a part that predicted a deviation from the correct answer value immediately before or after the maximum inspiration and maximum expiration. However, at most it is up to about 2 mm within the planned target volume for the 1.5-second prediction for a tumor moving about 16 mm, which would be an acceptable level.

**Figure 4:**
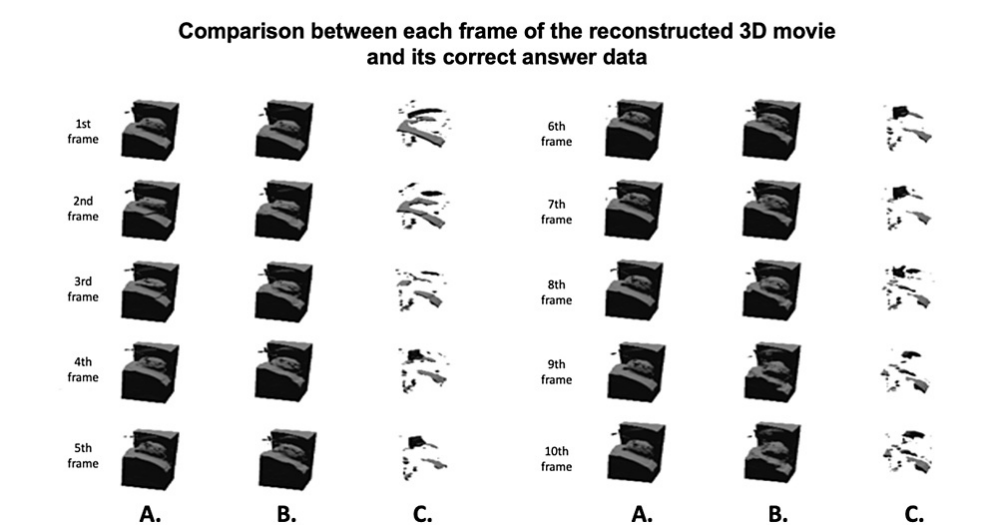
(A) Correct image. (B) Reconstructed image. (C) Difference image.

Figure 4 shows a comparison between each frame of the 3D movie of the tumor periphery reconstructed from the predicted 3D movement amount and its correct answer data (by OF). Immediately after the maximum inspiration (first and second frames), there is some deviation in the height of the diaphragm, but it can be seen that the tumor part can be predicted and reconstructed with almost no error.

From this result, it was shown that it can be effectively applied to both tracking and interception irradiation techniques in radiation therapy for lung cancer.

Real-time verification

The goal of this study is to confirm the location of the tumor from 4D-reconstructed MRI and to irradiate the confirmed location without delay. Therefore, we examined the imaging time of cine-MRI, the time from acquisition of cine-MRI to reconstruction to 4D MRI, the delay time from Beam-on to irradiation of the tumor, and the calculation time for future prediction of the tumor movement amount and direction, and we verified the real-time irradiation.

The results are shown in Table 2. The calculated time shown in Table 2 is the calculated time to 4D MRI reconstruction around the tumor of patient 5. The average of the calculation times for five times was calculated and verified.

**Table 2:** Algorithm calculation result (per frame) MRI, magnetic resonance imaging; LSTM, long short-term memory

Section 1		Section 2	Section 3			Total time	
Cine-MRI	Convert from MRI to CT image	Make input data	3D movement amount prediction by LSTM to 4D MRI reconstruction	Beam-On to irradiation to the tumor	Others (monitor output, etc.)		
							0.25 seconds	0.005 seconds	0.0003 seconds	0.42 seconds	0.5 seconds	α seconds	1.1753+α seconds	

From Table 2, the time required from the acquisition of cine-MRI to the irradiation of the tumor area is delayed by about 1.2 seconds per frame. In addition to this, the output time to the monitor in the treatment room will be added. In this study, we succeeded in predicting the amount of movement 1.5 seconds (six frames) ahead. Therefore, while observing the 3D tumor contour with 4D MRI, accurate irradiation to the irradiation area is realized without delay.

## Discussion

Future prediction of 3D movement by LSTM

Data on changes in the respiratory cycle during breathing were also used to input the verification data, and predictions can be made corresponding to those changes. However, we have not learned the rapid changes in respiratory dynamics, and we think that it is not effective at this time to learn them. Also, if the respiratory cycle is constant, it is considered possible to predict and reconstruct the amount of movement more accurately. Therefore, when adapting this algorithm during treatment, it is considered that something like a breathing guidance video for stabilizing the patient's breathing is effective.

A 4D MRI reconstruction from cine-MRI

In the present circumstances, we do not have 4D MRI data and thus we cannot perform quantitative evaluation. However, reasonable results have been obtained for respiratory cycle, amplitude, and tumor contour. This is considered to indicate that both modules in the algorithm proposed this time are working effectively. At present, the number of patient data is limited, and therefore it has not been possible to verify a sufficient number of patients. Therefore, it is necessary to increase the number of patients in the future.

Real-time performance

In this study, we evaluated real-time performance using MRI imaging time, irradiation delay, reconstruction time in the proposed algorithm, and future predictions for it. As shown in Table 2, in this study, six frames (1.5 seconds) were predicted considering the additional time that would occur during clinical adaptation. Since the time from acquisition of cine-MRI to irradiation of the tumor varies depending on the size of the reconstructed part (tumor peripheral part) and the irradiation method, we will need to adjust the number of frames and the reconstructed range to be actually predicted when we adapt to the actual device. In addition, although this study was conducted using the MRIdian cobalt system, the imaging speed and device delay will change when other systems are used.

The results drawn throughout this study are shown below in comparison with the research objectives and associated functional requirements.

・Conversion from MRI to CT image while maintaining high contrast

We have succeeded in creating a conversion network from MRI, which is a relative value image, to CT image, which is an absolute value image, and this conversion is normally impossible to calculate. In addition, by creating training data with adjusted image gradation degree, high-speed conversion was realized while maintaining the contrast ability required by this algorithm.

・Estimating tumor 3D movement by breathing and reconstructing 3D movies from cine-MRI

It was achieved by discovering that there is a correlation between the change in pixel value of a 2D image and the amount of movement in 3D and creating a network that connects them. Also, we developed a program to reconstruct a 3D movie from a predicted 3D movement and a reference 3D image.

・Future prediction of tumor migration amount for MRI imaging time and device delay time

By using LSTM and optimal parameter setting, it is possible to predict the future of time series data, which succeeded in predicting the amount of 3D movement 1.5 seconds ahead.

## Conclusions

Based on the above, we have achieved the purpose of this research, that is, proposal of algorithm and method for achieving high accuracy of tracking and gating irradiation technology for mobile tumors using MR-linac.

By using this method, it is possible to draw and confirm the current position of the tumor that moves by breathing during radiation therapy in 3D, and it will help to achieve high precision radiotherapy with less unnecessary exposure. It is recommended to create a movement prediction model (Module#1) for each patient because the movement of organs (tumors) in breathing has patient-specific characteristics depending on the patient. The accuracy of conversion from MR images to CT images can be expected to be further improved by adding training data. However, with this method, the current conversion accuracy will be sufficient.
